# Density Functional Theory Calculation May Confirm Arsenic–Thiol Adhesion as the Primary Mechanism of Arsenical Toxicity

**DOI:** 10.1021/acsomega.3c09269

**Published:** 2024-03-13

**Authors:** Meng-Han Tsai, Ying-Ting Lin

**Affiliations:** † Department of Biotechnology, College of Life Science, Kaohsiung Medical University, Kaohsiung 80708, Taiwan; ‡ Drug Development & Value Creation Research Center, Kaohsiung Medical University, Kaohsiung 807, Taiwan

## Abstract

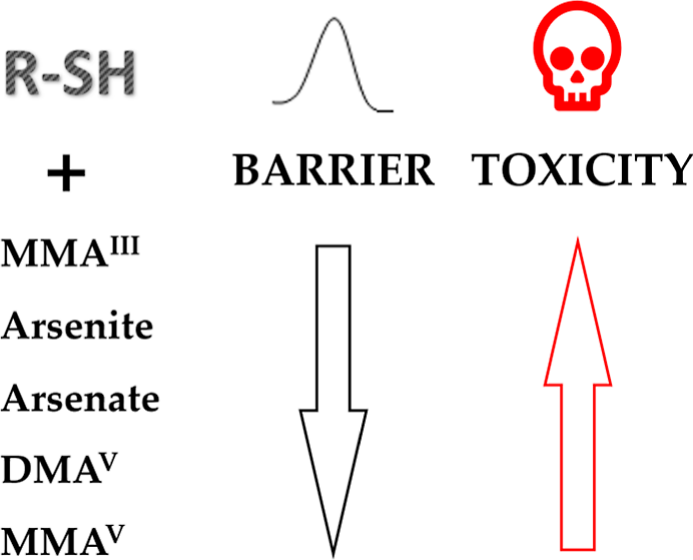

Previously, it was believed that methylation was the body’s primary method to detoxify inorganic arsenic. However, recent research has shown that the metabolized intermediate known as MMA^III^ is more toxic than arsenite and arsenate, contradicting a previous understanding. Another important question arises: is arsenical toxicity truly caused by arsenic binding to proteins through arsenic thiol adhesion? Based on the toxicity order of the experiment, with MMA^III^ being the most toxic, followed by arsenite, arsenate, DMA^V^, and MMA^V^, density functional theory (DFT) calculations can provide a straightforward assessment of this issue. Our practice captures all the transition states associated with a specific imaginary-frequency vibration mode, including proton transfer and simultaneous departure of leaving group. We have obtained the energy barriers for five arsenicals reacting with thiol, alcohol, and amine separately. In addition to energetic favorability, the following are the energy barriers for arsenic’s reaction with thiol ranked from low to high: MMA^III^ (25.4 kcal/mol), arsenite (27.7 kcal/mol), arsenate (32.8 kcal/mol), DMA^V^ (36.2 kcal/mol), and MMA^V^ (38.3 kcal/mol). Results show that the toxicity of arsenicals is mainly caused by their reaction with thiol rather than with alcohol or amine, as supported by the trend of decreasing toxicity and increasing energy barriers. Thus, this DFT calculation may confirm the paradigm that arsenic–thiol adhesion is the primary cause of arsenic toxicity in the body.

## 1 Introduction

Arsenic, a heavy metal pollutant, is ubiquitous in the environment, often combining with other metals to form mineral rocks that transform into dust. It can also dissolve in rainwater, rivers, and groundwater, entering the water cycle and posing a threat to organisms.^1,2^ Arsenic poisoning, caused by the consumption of drinking water contaminated with arsenic, can lead to acute consequences such as peripheral neuropathy, chronic lung disease, increased incidence of liver and cardiovascular disease, as well as chronic consequences such as skin, lung, liver, kidney, and bladder cancers.^3^ To address this issue, the World Health Organization established a maximum permissible limit of 10 μg/L for arsenic ingestion through drinking water in 2011.^4^ The body processes arsenic mainly through two metabolic processes: the reduction of pentavalent arsenic to trivalent arsenic and the methylation of trivalent arsenic to pentavalent arsenic.^5^ Glutathione and reductase enzymes dominate the reduction of pentavalent arsenic compounds,^6−8^ while *S*-adenosylmethionine and methyltransferase are responsible for the methylation of trivalent arsenic compounds to pentavalent ones.^9^

Whereas the two metabolized products, monomethylarsenic acid (MMA^V^) and dimethylarsenic acid (DMA^V^), have demonstrated low toxicity in acute lethality assays, and the process of methylation of arsenicals was previously believed to be a detoxification mechanism.^10,11^ However, the extraction of another metabolized product, monomethylarsonous acid (MMA^III^), from human urine has indicated that MMA^III^ is more toxic than arsenite.^12^ As a result, cytotoxicity studies have been conducted to determine the relative toxicity of various arsenicals. A toxicity ranking (MMA^III^ > arsenite > arsenate > DMA^V^ = MMA^V^) has been proposed.^13^ Several possible carcinogenic mechanisms have also been suggested for arsenical, based on in vivo and in vitro experiments, which yielded the same order of toxicity as stated above.^14^ The toxicity of monomethylarsonous acid (MMA^III^) is greater than those of arsenite and arsenate, indicating that the methylation process may not be solely a detoxification mechanism.

Arsenical toxicity is a complex phenomenon that involves multiple biological pathways and triggers skepticism that the arsenic–thiol adhesion might not be the sole reason to account for the various harmful effects of arsenic. The arsenic–thiol reaction can result in the depletion of intracellular glutathione, a major thiol-containing antioxidant, and subsequent oxidative stress. Arsenical has also the ability to disrupt cellular signaling,^15,16^ and impair mitochondrial function.^13,17^ Moreover, arsenical is also suggested to alter the expressions of other cellular components, such as lipids^18^ and nucleic acids,^19,20^ and affect various biochemical and physiological processes. Thus, a question is then raised against the current conception: does arsenical toxicity primarily come from binding of arsenic to protein cysteine, i.e., arsenic thiol adhesion?

The density functional theory (DFT)^21^ is a computational method used in quantum mechanics to investigate the electronic structure of molecules and materials. It can calculate various properties of molecules including their energy, geometry, and electronic structure. A DFT calculation may provide a simple assessment of the above question by checking if the somatic toxicity can be explained by the DFT-calculated reaction energy profile (REP), of which one important feature is the transition state (TS) energy barrier. In this study, we calculated all REPs between arsenicals and methanethiol, methanol, and methylamine. Our goal was to identify which functional groups arsenic primarily interacts with to cause somatic toxicity by observing model systems with the five mentioned arsenicals reacting with three main functional groups: thiol, alcohol, and amine. All the calculated TSs feature the only imaginary frequency where a vibration mode of proton-transfer couples with the concerted departure of the leaving group. Through DFT calculations, we have discovered that the energy barriers of thiol reactions are increasing while toxicity is decreasing. This trend holds true for the five arsenicals reacting with thiol, as opposed to arsenic reactions with alcohol or amine. These analyses convey that arsenical toxicity is mainly caused by the reaction of arsenic with thiol. Therefore, arsenic–thiol adhesion is the primary cause of arsenical toxicity in the body.

## 2 Materials and Methods

**Figure 1:**
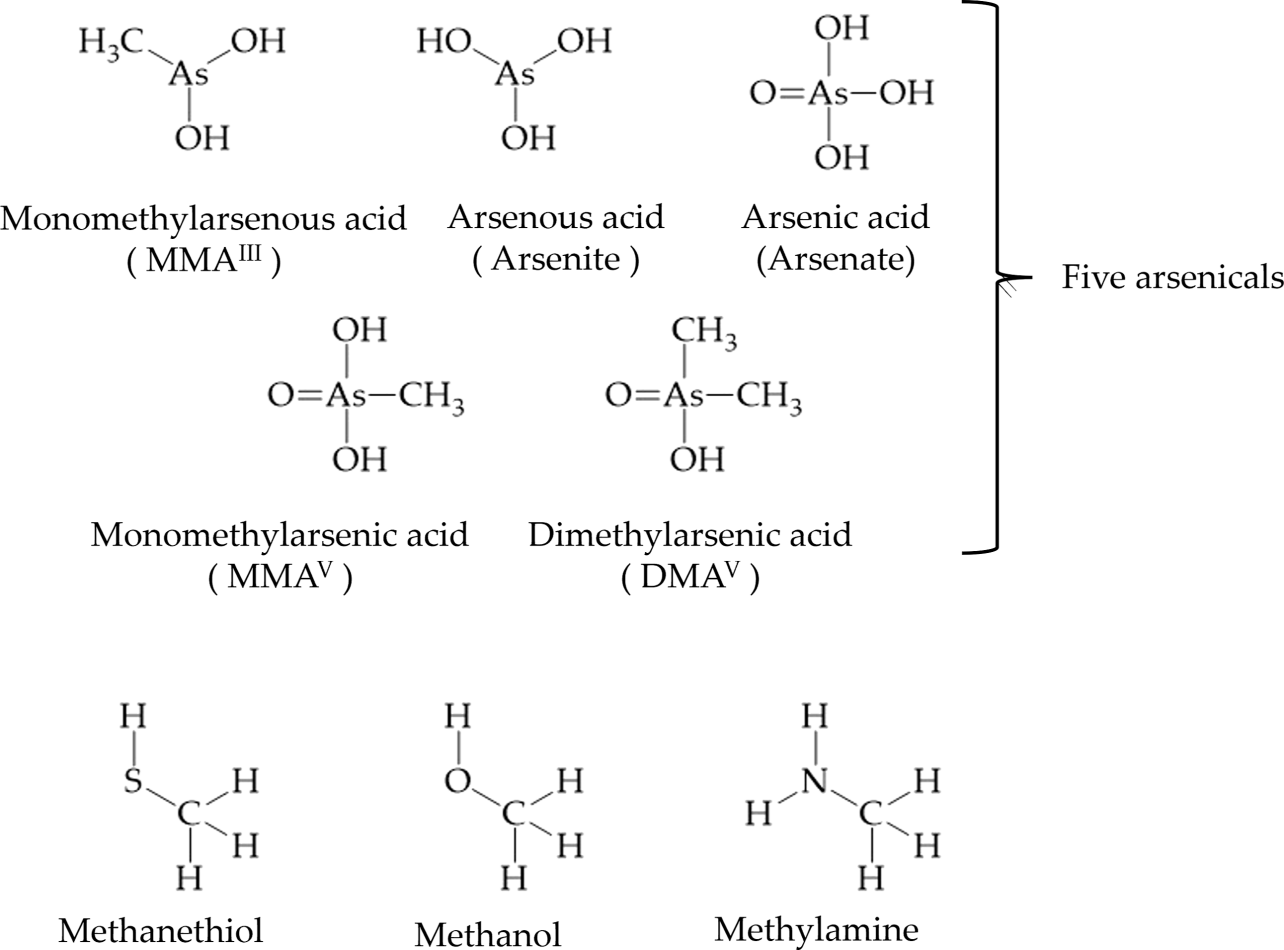
Five arsenicals and three molecules, methanethiol, methanol, and methylamine, as reactants are used in the model systems of the DFT calculation.

Five arsenicals, one thiol molecule, one alcohol molecule, and one amine molecule as reactants are used in the model systems of the DFT calculation.^21^ The five arsenicals comprise MMA^III^ [monomethylarsenous acid, CH_3_As^III^(OH)_2_], arsenite [arsenous acid, As^III^(OH)_3_], arsenate [arsenic acid, As^V^O(OH)_3_], MMA^V^ [monomethylarsenic acid, CH_3_As^V^O(OH)_2_], and DMA^V^ [dimethylarsenic acid, (CH_3_)_2_As^V^O(OH)]. The thiol molecule is methanethiol (HSCH_3_), the alcohol molecule is methanol (HOCH_3_), and the amine molecule is methylamine (H_2_NCH_3_) as shown in Figure 1. To analyze the interactions between arsenicals and thiol, alcohol, and amine molecules, we utilized DFT to calculate their energy profiles. First, we carefully determine the TS,^22^ then optimize the reactants and products. This TS should exhibit a proton transfer mechanism with an imaginary-frequency vibrational mode,^21^ coupled with the simultaneous departure of a leaving group. To begin, we utilized this TS as our initial reference and brought the proton closer to the reactant. Afterward, we conduct geometry optimization through DFT to achieve the desired reactant. We move the proton near the product, and the optimization will produce the desired products. The section of results and discussion will detail more information, using MMA^III^ and methanethiol as examples. We can use the same calculation practice to obtain all of the energy profiles through this unified process of DFT geometry optimization. The DFT calculations (using uB3LYP functional method,^23^ charge = 0, and spin = 1) were mainly carried out using the Gaussian 16 software package^24^ with 6-31G(d) basis set.^25,26^ All structures for the molecules under investigation were generated using GaussView 6.0.^27^ The energy unit used to display the REP is kcal/mol in the calculated results. Notice that the energy barrier is the TS energy minus the reactant energy and the reaction energy is the product energy minus the reactant energy.

## 3 Results and Discussion

### 3.1 Capture Transition State with Proton Transfer Coupled with the Simultaneous Departure of Leaving Group

**Figure 2:**
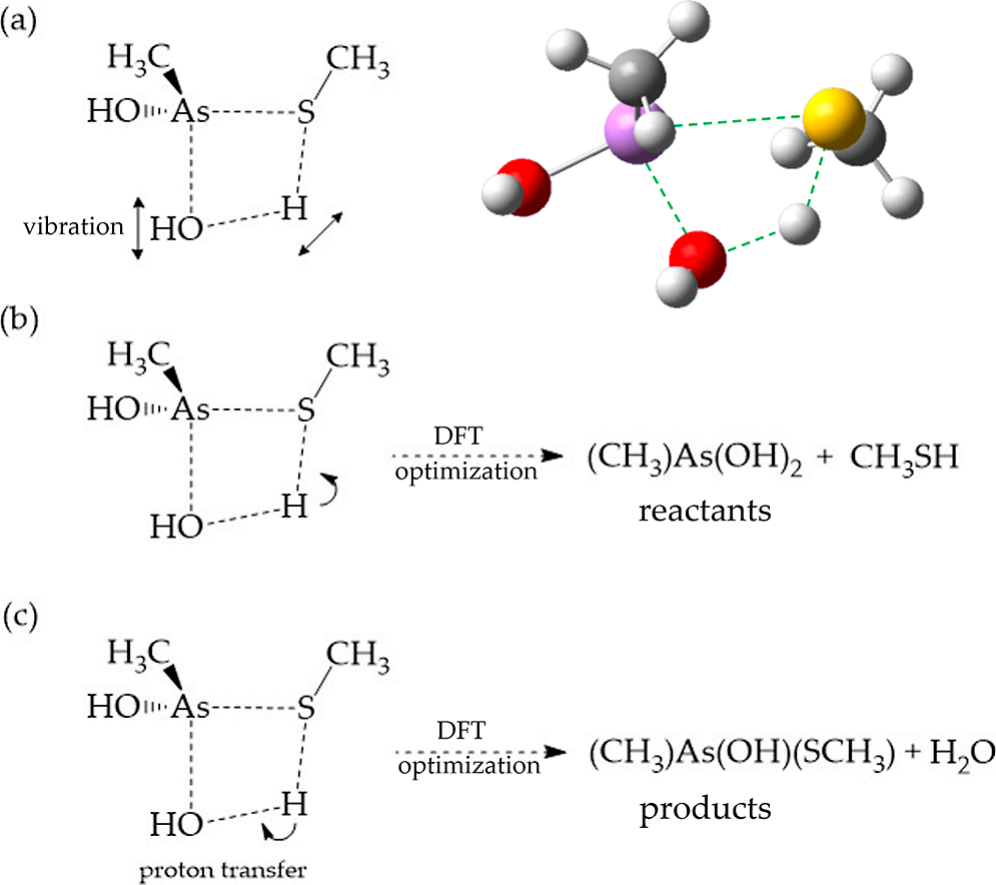
(a) The captured TS between MMA^III^ and methanethiol with an imaginary-frequency vibrational mode featuring proton transfer and the departure of the hydroxyl group. We used this TS geometry as an initial point for further geometry optimization. The right panel shows a stereo view of the TS. (b) Relocating the proton close to the sulfur atom of methanethiol in the DFT geometry optimization calculation will yield the reactants. (c) Relocating the proton close to the oxygen atom of the hydroxyl group will yield the products.

**Figure 3:**
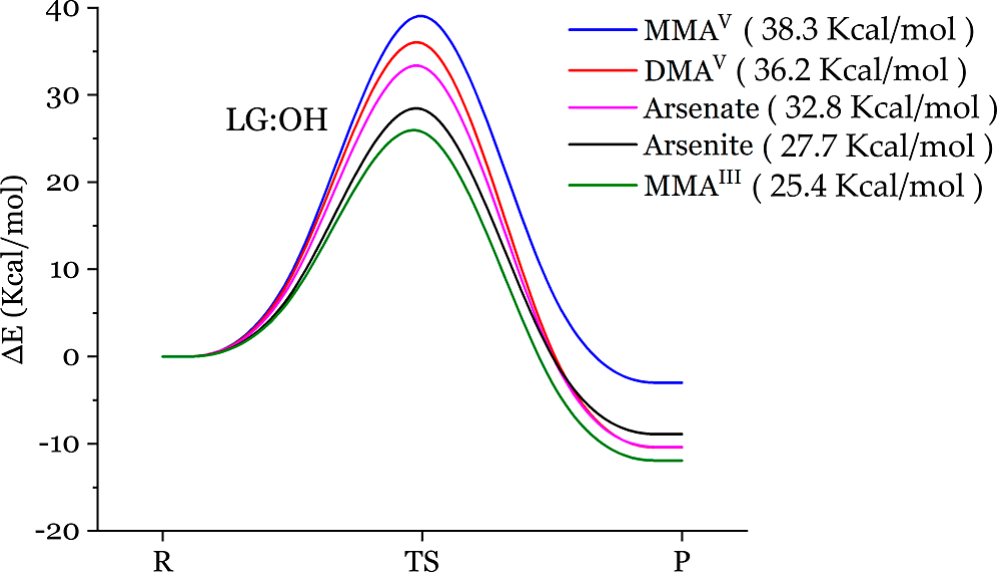
REPs for the reactions between the five arsenicals and methanethiol with leaving the hydroxyl group. The energy barriers of arsenical reacting with thiol, ranked from low to high, are MMA^III^ (25.4 kcal/mol) < arsenite (27.7 kcal/mol) < arsenate (32.8 kcal/mol) < DMA^V^ (36.2 kcal/mol) < MMA^V^ (38.3 kcal/mol). LG denotes the leaving group.

Arsenical reacting with methanethiol at various orientations is likely to cause different TSs, probably leading to different products. Here, we propose to use a specific TS as the initial structure point for all of the geometry optimization along the reaction pathway. It is possible to avoid errors in optimizing the geometry of random initial structures for reactants and products. Take the reactants, MMA^III^, and methanethiol, as an example. For instance, in Figure 2a, in the captured TS state, the four atoms, including arsenic, hydroxyl oxygen, thiol sulfur, and thiol hydrogen, form a quadrilateral of a TS at an energy high. After the DFT geometry optimization for the TS structure, we can observe that an imaginary-frequency vibrational mode has a proton transfer between the sulfur atom of methanethiol and the oxygen atom of the hydroxyl group, coupled with the departure of the hydroxyl group. In Figure 2b, the captured TS state serves as the starting point for further calculations of the structures of the reactants and products. By bringing the proton a bit closer to the sulfur atom of thiol in the TS initial structure, a DFT geometry optimization calculation can obtain the optimized structures and energies of the two reactants, MMA^III^ and methanethiol. In Figure 3c, by moving the proton a bit closer to the oxygen of the arsenic hydroxyl in the TS initial structure, the DFT geometry optimization calculation can find the optimized structures and energies of the arsenic product complex and water. To maintain consistency, we propose creating a uniform calculation practice for all REPs^22^ involving the five arsenicals reacting with methanethiol, alcohol, and amine in a vacuum. By doing so, we can eliminate the possibility of various TS states that may occur due to different reaction orientations. To properly capture TSs, they must be linked to a specific vibration mode in a quadrilateral orbit of the related four atoms. This imaginary-frequency vibration mode features proton transfer and the simultaneous departure of the leaving group. This way allows us to accurately determine the energy barriers for reactions involving arsenic and thiol, alcohol, or amine. In order to accurately identify barrier trends, it is crucial that we maintain consistency in both our method and our approach to achieve consistent results.

### 3.2 Energy Profile for Arsenicals Reacting with Methanethiol Leaving Hydroxyl Group

As aforementioned in Figure 2, the four atoms, including arsenic, hydroxyl oxygen, thiol sulfur, and thiol hydrogen, can form a quadrilateral at an energy high. The TS recognized by a specific imaginary-frequency vibrational mode features a proton transfer and the simultaneous departure of the hydroxyl group. We first capture such TS structures and then further geometry-optimize the reactants and products by relocating the TS proton-transferring proton a bit closer to the sulfur, oxygen, or carbon atom. Therefore, related arsenical reactants and products can be obtained. Table 1 lists the energy barriers and reaction energies for the reactions between the five arsenicals, MMA^III^, arsenite, arsenate, DMA^V^, MMA^V^, and the thiol, methanethiol, with two different leaving groups, hydroxyl or methyl. The order of these energy barriers remains unchanged even after some minor basis set corrections (6-31G(d,p)) and thermal corrections (Gibbs free energy). In Figure 3, one can clearly see that the energy barrier of arsenic reacting with thiol, ranked from low to high, are MMA^III^ (25.4 kcal/mol) < arsenite (27.7 kcal/mol) < arsenate (32.8 kcal/mol) < DMA^V^ (36.2 kcal/mol) < MMA^V^ (38.3 kcal/mol). We also note that all of the reaction energy values are negative, indicating that these reactions are energetically favorable. The lower energy barrier between arsenicals and methanethiol implicates the high biological toxicity since such arsenicals more easily form complexes with cysteine. The side chain of cysteine includes a thiol functional group. This rank order of the energy barriers almost concurs with the confirmed toxicity rank order, MMA^III^ > arsenite > arsenate > DMA^V^ = MMA^V^,^13^ except for the DMA^V^ and MMA^V^. Since DMA^V^ (36.2 kcal/mol) has a bit lower energy barrier than MMA^V^ (38.3 kcal/mol), DMA^V^ should be less toxic than MMA^V^ rather than having a similar level of toxicity. Yet, MMA^V^ with two hydroxyl groups has more opportunity to bind to cysteine, increasing its toxicity. Therefore, if we balance out the two opposing factors, the toxicity of DMAV and MMAV may be similar. To sum it up, energetic favor and the concurrent trend of rank suggest that arsenical toxicity in the body may originate from the arsenic–thiol adhesion, i.e., the arsenic–sulfur bond forming.

**Table 1:** Energy Barriers and Reaction Energies for the Reactions between the Five Arsenicals and Methanethiol with Varying Leaving Groups, Hydroxyl, or Methyl^a^

arsenicals	leaving group (LG)	energy barrier (Δ*E*a)	reaction energy (Δ*E*r)
MMA^III^	OH	25.4, 23.9^b^, 25.2^c^	–11.9, −11.5^b^, −8.3^c^
	CH_3_	54.6	–16.1
arsenite	OH	27.7, 25.9^b^, 28.2^c^	–8.9, −10.4^b^, −5.2^c^
arsenate	OH	32.8, 32.7^b^, 34.4^c^	–10.4, −10.7^b^, −7.6^c^
DMA^V^	OH	36.2, 34.9^b^, 37.5^c^	–10.4, −12.5^b^, −7.4^c^
	CH_3_	66.7	–14.1
MMA^V^	OH	38.3, 37.3^b^, 38.0^c^	–3.0, −4.5^b^, −1.8^c^
	CH_3_	69.5	–6.7

a The DFT calculations used the uB3LYP method and the 6-31G(d) basis set.

b Using 6-31G(d,p) basis set.

c Using 6-31G(d,p) basis set and thermal correction, i.e. Gibbs free energy.

### 3.3 Energy Profile for Arsenicals Reacting with Methanethiol Leaving Methyl Group

**Figure 4:**
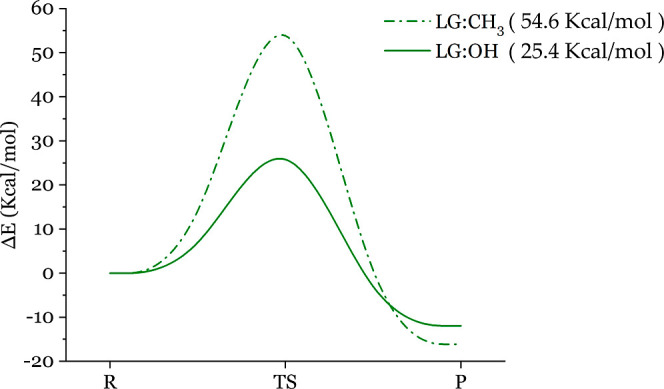
REPs for the reactions between MMA^III^ and methanethiol with leaving the hydroxy group. The energy barrier of arsenic reacting with methanethiol, leaving the methyl group, is 54.6 kcal/mol. While the energy barrier of arsenic reacting with methanethiol leaving the hydroxyl group is 25.4 kcal/mol.

Arsenical approaching methanethiol at different orientations may produce different TSs, leading to different products. Again, we take MMA^III^ and methanethiol leaving “methyl group” as another example. As shown in Supporting Information Figure S1, results show that four atoms, including arsenic, “methyl carbon”, thiol sulfur, and thiol hydrogen, form a similar quadrilateral of a TS at an energy high. The four atoms, including arsenic, methyl carbon, thiol sulfur, and thiol hydrogen, can form a quadrilateral at an energy high. The TS has an imaginary frequency caused by a proton transfer vibration mode and here the simultaneous departure of “the methyl group”. We again capture the TS structures and then further geometry-optimize the reactants and products by locating the TS proton-transferring proton near the sulfur or carbon atom. The relative arsenical reactants and products can be obtained. In Figure 4, one can clearly see in the REP that the energy barrier of arsenic reacting to thiol with the departure of the methyl group is 54.6 kcal/mol. The energy barrier of arsenic reacting with thiol leaving the hydroxyl group is 25.4 kcal/mol. The energy barrier of leaving the methyl group is more than double the barrier of leaving the hydroxyl group. In Table 1, we can clearly see that the energy barriers of leaving a methyl group for DMA^V^ and MMA^V^ are 66.7 and 69.5 kcal/mol, nearly double those of leaving a hydroxyl group. It is more difficult for a methyl group to detach compared to a hydroxyl group due to higher energy barriers. The findings indicate that it is unlikely for arsenical to leave a methyl group during its interaction with cysteine thiol.

### 3.4 Energy Profile for Arsenicals Reacting with Methanol Leaving Hydroxyl Group

**Figure 5:**
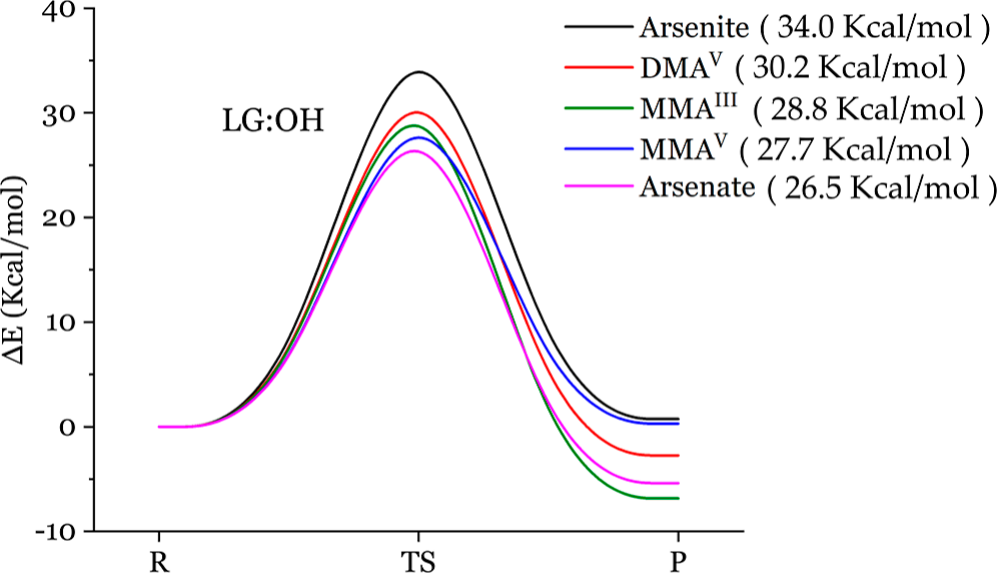
REPs for the reactions between the five arsenicals and methanol with leaving a hydroxyl group. The energy barriers of arsenical reacting with methanol, ranked from low to high, are arsenate (26.5 kcal/mol) < MMA^V^ (27.7 kcal/mol) < MMA^III^ (28.8 kcal/mol) < DMA^V^ (30.2 kcal/mol) < arsenite (34.0 kcal/mol). LG denotes leaving group.

To see if some other different mechanisms can cause similar toxicity rank order of arsenicals, we examined the REP between arsenical and methanol. This model system represents the interaction between arsenic and oxygen atom rather than sulfur atom. In this system, the four atoms, arsenic, hydroxyl oxygen, methanol oxygen, and methanol hydrogen, form a quadrilateral at an energy high. The TS recognized by a specific imaginary-frequency vibration mode features a proton transfer and the simultaneous departure of the hydroxyl group as shown in the Supporting Information Figure S2. We captured the TS structures and then further geometry-optimized the reactants and products by bringing the TS proton-transferring proton a bit near the reactant or product oxygen atom. The arsenical reactants and products can be obtained. Therefore, Table 2 lists the energy barriers and reaction energies for the reactions between the five arsenicals, MMA^III^, arsenite, arsenate, DMA^V^, MMA^V^, and the thiol, methanol, leaving the hydroxyl group. Notably, the reaction energy values are near and close to zero, indicating that these reactions are not favorable in terms of energy. In Figure 5, one can see that the energy barrier of arsenic reacting with alcohol, ranked from low to high, are arsenate (26.5 kcal/mol) < MMA^V^ (27.7 kcal/mol) < MMA^III^ (28.8 kcal/mol) < DMA^V^ (30.2 kcal/mol) < arsenite (34.0 kcal/mol). In this case, the lower energy barrier between arsenicals and methanol would associate the higher biological toxicity if arsenical can form a complex with, say, serine. The side chain of serine includes an alcohol functional group. However, this order of arsenical energy barriers is not consistent with the experimental toxicity order, MMA^III^ > arsenite > arsenate > DMA^V^ = MMA^V^. Overall, energetic disfavor and the disagreeing trend of rank suggest that arsenical toxicity in the body may not come from the arsenic-alcohol adhesion, i.e., not the arsenic–oxygen bond forming.

**Table 2:** Energy Barriers and Reaction Energies for the Reactions between the Five Arsenicals and Methanol with the Departure of the Hydroxyl Groups^a^

arsenicals	leaving group (LG)	energy barrier (Δ*E*a)	reaction energy (Δ*E*r)
MMA^III^	OH	28.8	–6.9
arsenite	OH	34.0	0.7
arsenate	OH	26.5	–5.4
DMA^V^	OH	30.2	–2.7
MMA^V^	OH	27.7	0.3

a The DFT calculations used the uB3LYP method and the 6-31G(d) basis set.

### 3.5 Energy Profile for Arsenicals Reacting with Methylamine Leaving Hydroxyl Group

**Figure 6:**
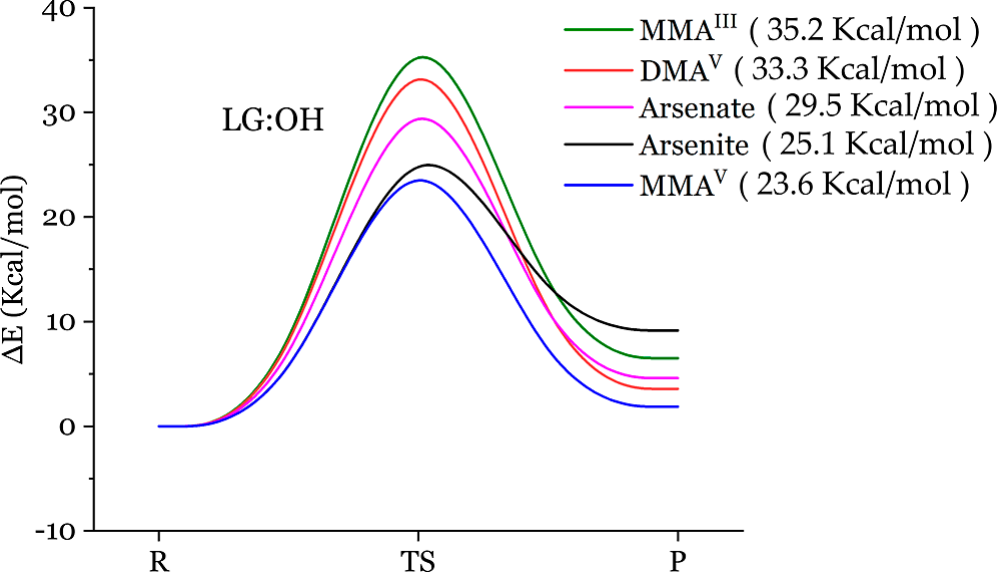
REPs for the reactions between the five arsenicals and methylamine with the leaving hydroxyl group. The energy barriers of arsenical reacting with methylamine, ranked from low to high, are MMA^V^ (23.6 kcal/mol) < arsenite (25.1 kcal/mol) < arsenate (29.5 kcal/mol) < DMA^V^ (33.3 kcal/mol) < MMA^III^ (35.1 kcal/mol). LG denotes leaving group.

Again, to see if other mechanisms can cause similar toxicity rank orders of arsenicals, we inspected the REP between arsenical and methylamine. In this case, the four atoms, arsenic, hydroxyl oxygen, methylamine nitrogen, and methylamine hydrogen, form a quadrilateral at an energy high. The TS once more recognized by an imaginary-frequency vibration mode features a proton transfer and the simultaneous departure of the hydroxyl group as shown in the Supporting Information Figure S3. We captured the TS structures likewise and geometry-optimized the reactants and products by moving the TS proton-transferring proton a bit near the reactant nitrogen atom or product oxygen atom. The related arsenical reactants and products can be obtained. Therefore, Table 3 lists the energy barriers and reaction energies for the reactions between the five arsenicals, MMA^III^, arsenite, arsenate, DMA^V^, MMA^V^, and the amine, methylamine, leaving the hydroxyl group. Additionally, the values of reaction energy are positive, implying that these reactions are energetically disfavored. In Figure 6, one can see that the energy barrier of arsenic reacting with methylamine, from low to high, is MMA^V^ (23.6 kcal/mol) < arsenite (25.1 kcal/mol) < arsenate (29.5 kcal/mol) < DMA^V^ (33.3 kcal/mol) < MMA^III^ (35.1 kcal/mol). The lower energy barrier between arsenicals and methylamine would associate the higher biological toxicity if arsenical can form a complex with, say, lysine. The side chain of lysine contains the functional group of a primary amine. However, this order of arsenical energy barriers contradicts the experiment rank order of toxicity, MMA^III^ > arsenite > arsenate > DMA^V^ = MMA^V^. Again, the increased level of positive reaction energy and the conflicting hierarchy indicate that arsenical toxicity in the body does not come from the arsenic-amine adhesion, i.e., not the arsenic–nitrogen bond forming.

**Table 3:** Energy Barriers and Reaction Energies for the Reactions between the Five Arsenicals and Methylamine with the Departure of the Hydroxyl Group^a^

arsenicals	leaving group (LG)	energy barrier (Δ*E*a)	reaction energy (Δ*E*r)
MMA^III^	OH	35.2	6.5
arsenite	OH	25.1	9.2
arsenate	OH	29.5	4.6
DMA^V^	OH	33.3	3.6
MMA^V^	OH	23.6	1.9

a The DFT calculations used the uB3LYP method and the 6-31G(d) basis set.

Finally, it is worth highlighting that refining the accuracy of reaction energies calculated through DFT is achievable by employing more precise DFT functionals tailored to specific reaction conditions. However, the pursuit of ideally accurate values may curtail our ability to explore and compare results with larger systems, especially as sophisticated methods become more susceptible to breakdowns in expansive biological systems. Consequently, our research strategically focuses on capturing the right proton-transfer TSs and observing trends derived from fundamental DFT methodology, forgoing the use of multiple correction methods in both ambient conditions and solutions. This approach will allow us to delve into a broader spectrum of future systems, including larger ones, without the necessity for postprocessing corrections. Through this deliberate choice, we derive valuable insights into the correlation between the arsenical–thiol activation energies and arsenic toxicity. As shown in Table 1 with additional basis set and thermal corrections, we maintain the belief that any more systematic, higher-level corrections applied later will not significantly alter the observed trends in this study.

## 4 Conclusions

When arsenical reacts with methanethiol, the reaction energy values are all negative, suggesting that these reactions are energetically favored. The energy barriers are ranked from low to high as MMA^III^ < arsenite < arsenate < DMA^V^ < MMA^V^. On the other hand, when arsenical reacts with methanol, the reaction energy values are close to zero, proposing that these reactions are not energetically favored. The energy barriers are ranked from low to high as arsenate < MMA^V^ < MMA^III^ < DMA^V^ < arsenite. When arsenical reacts with methylamine, the reaction energy values are all positive, indicating that these reactions are energetically disfavored. The energy barriers are ranked from low to high as MMA^III^ < arsenite < arsenate < DMA^V^ < MMA^V^. Finally, in addition to energetic favorability, the rank order of the arsenical reaction with thiol is closely equivalent to the known toxicity rank order. This DFT calculation may confirm for the first time that the adhesion of arsenic to thiol may be the primary cause of arsenic toxicity.

## Supporting Information Available

Additional energy profiles of DFT calculation for more arsenical reactions, and all the Cartesian coordinates for all the transition states reacting with methanethiol, methanol, and methylamine (PDF)
