# Supplementary material for: Density Functional Theory Calculation May Confirm Arsenic–Thiol Adhesion as the Primary Mechanism of Arsenical Toxicity
Source: ACS Omega. 2024 Mar 13;9(12):13975–81. doi: 10.1021/acsomega.3c09269 (PMC10976359; doi:10.1021/acsomega.3c09269)
Supplement: Supplementary file 1 — ao3c09269_si_001.pdf [file ao3c09269_si_001.pdf]

Supporting Information:

A DFT Calculation May Confirm Arsenic-Thiol Adhesion as the Primary Mechanism of Arsenical Toxicity

Meng-Han Tsai [1], and Ying-Ting Lin [1,2]*

1. Department of Biotechnology, College of Life Science, Kaohsiung Medical University, Kaohsiung 80708, Taiwan

2. Drug Development & Value Creation Research Center, Kaohsiung Medical University, Kaohsiung 80708, Taiwan

Figure S1.

Figure S2.

Figure S3.

Table S1.

Table S2.

Table S3.

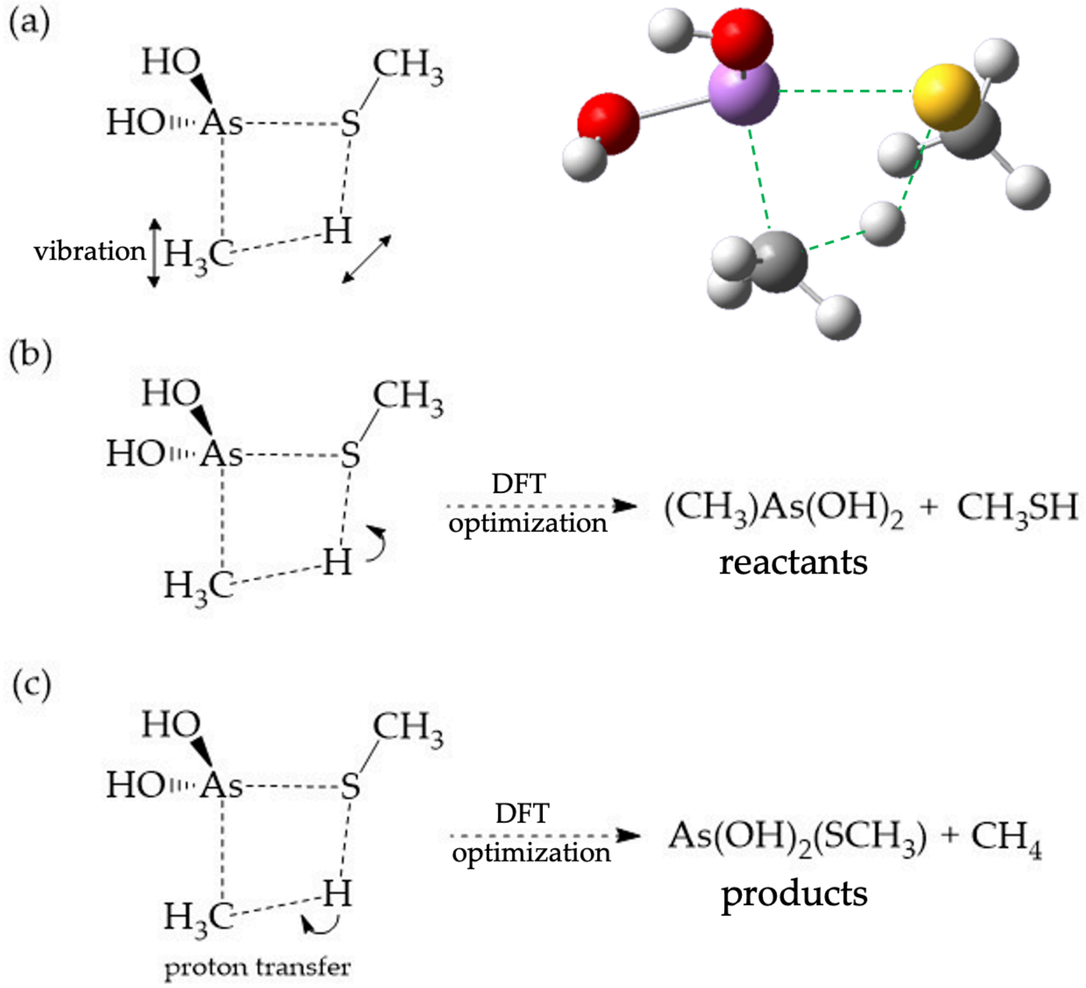

Figure S1. (a) The captured transition state between MMA^{III} and methanethiol with an imaginary-frequency vibrational mode featuring proton transfer and the departure of "the methyl group". We used this TS geometry as an initial point for further geometry optimization. The right panel shows a stereo view of the transition state. **(b)** Relocating the proton close to the sulfur atom of methanethiol in the DFT geometry optimization calculation will yield the reactants. **(c)** Relocating the proton close to the oxygen atom of the hydroxyl group will yield the products.

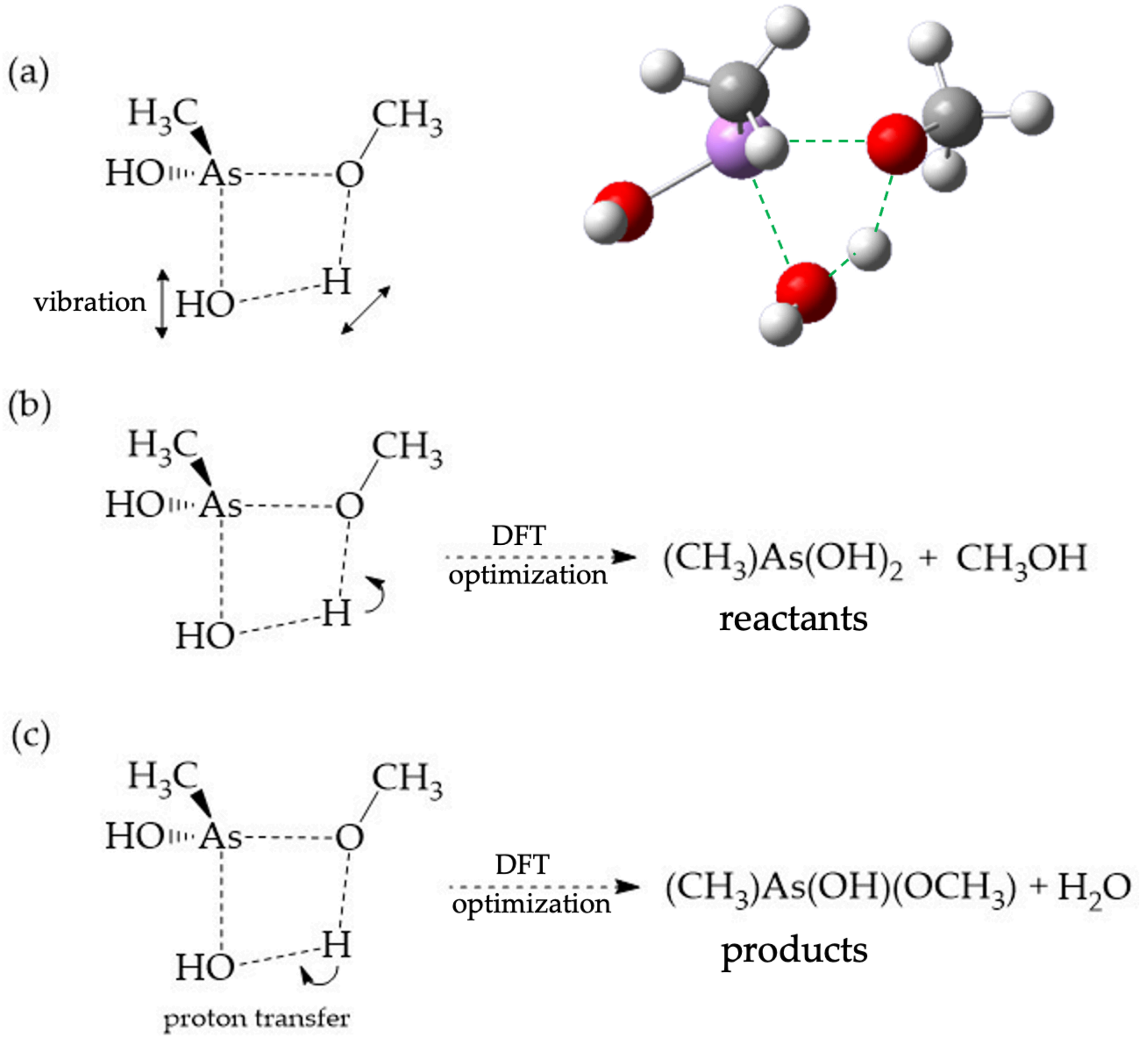

Figure S2. **(a)** The captured transition state between MMA^{III} and methanol with an imaginary-frequency vibrational mode featuring proton transfer and the departure of the hydroxyl group. We used this TS geometry as an initial point for further geometry optimization. The right panel shows a stereo view of the transition state. **(b)** Relocating the proton close to the oxygen atom of methanol in the DFT geometry optimization calculation will yield the reactants. **(c)** Relocating the proton close to the oxygen atom of the hydroxyl group will yield the products.

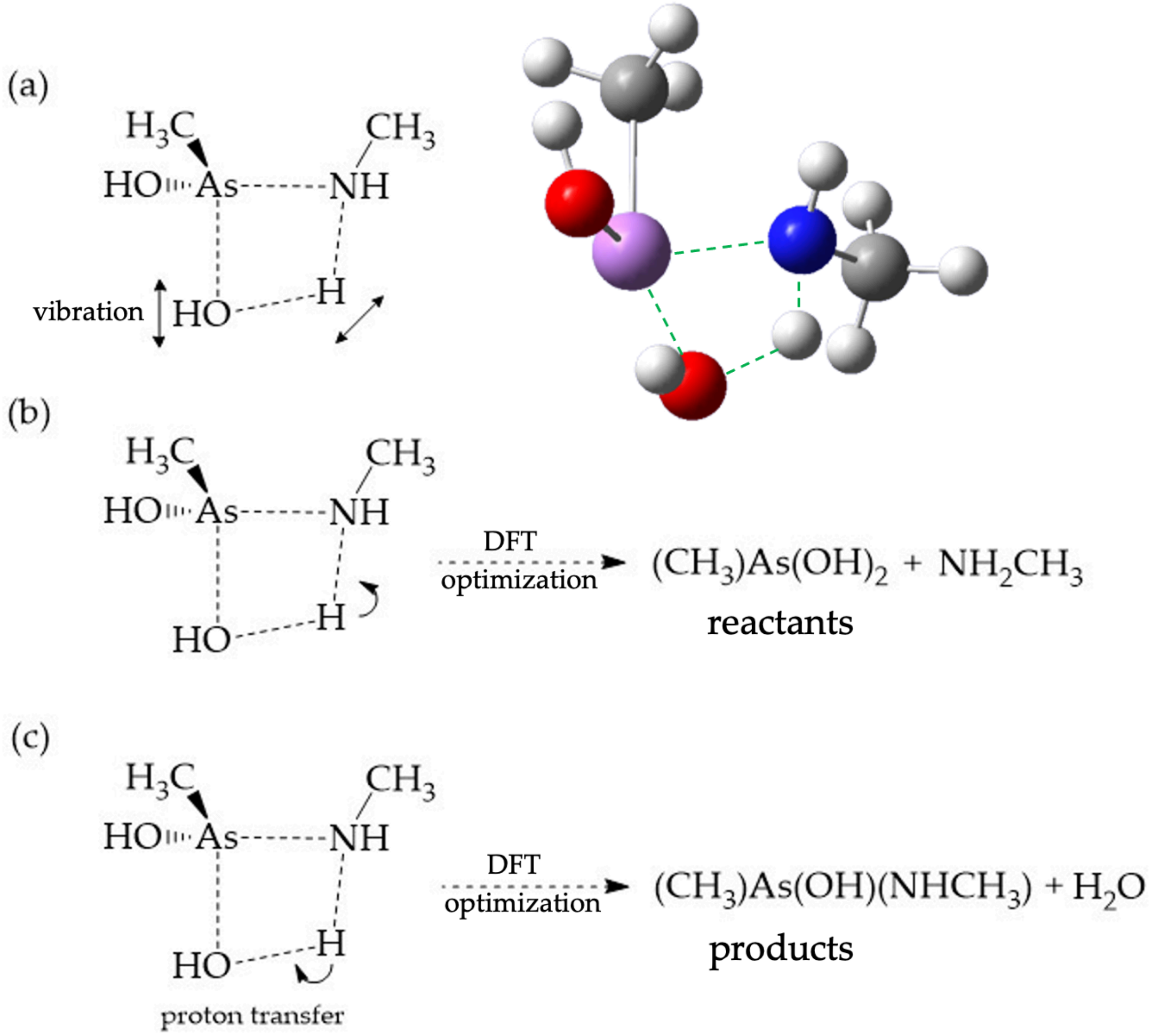

Figure S3. (a) The captured transition state between MMA^{III} and methylamine with an imaginary-frequency vibrational mode featuring proton transfer and the departure of the hydroxyl group. We used this TS geometry as an initial point for further geometry optimization. The right panel shows a stereo view of the transition state. **(b)** Relocating the proton close to the nitrogen atom of methylamine in the DFT geometry optimization calculation will yield the reactants. **(c)** Relocating the proton close to the oxygen atom of the hydroxyl group will yield the products.

Table S1. The cartesian coordinates for the transition states of Arsenicals reacting with methanethiol

Arsenical	Atom	Cartesian coordinates		
MMA^{III}	As	-0.68656500	-0.13434900	-0.42301900
	S	1.91995700	0.66484900	0.24780000
	C	2.77518700	-0.75862200	-0.55262000
	H	3.42730800	-0.38295600	-1.34538900
	H	3.38529700	-1.30090700	0.17443200
	H	2.05232300	-1.45105000	-0.99787900
	O	-2.33894000	-0.85707200	-0.12254200
	H	-2.90060100	-0.29707600	0.43844500
	O	-0.11405000	-0.85226800	1.42012700
	H	-0.68340100	-0.53711800	2.14694600
	C	-1.21132300	1.69778200	0.09657600
	H	0.82993800	-0.30162500	1.37362400
	H	-0.47830400	2.40401200	-0.29469600
	H	-2.19741700	1.90457900	-0.33094100
	H	-1.25706200	1.79784400	1.18586300
Arsenite	As	-0.65286800	-0.03248400	-0.41784000
	S	1.88725400	0.62049800	0.33808600
	C	2.65396800	-0.72532300	-0.65814400
	H	1.89553600	-1.30712100	-1.20011800
	H	3.32629200	-0.28208400	-1.39673000
	H	3.23302800	-1.40242300	-0.02431300

	O	-2.32049400	-0.75198900	-0.21570000
	H	-2.62810300	-0.69009500	0.70559200
	H	0.82954300	-0.38468000	1.33585500
	O	-1.09561900	1.57252100	0.20996600
	H	-2.06699500	1.59641700	0.26879300
	O	-0.21193300	-0.84778300	1.42056100
	H	-0.14017500	-1.81604400	1.32052900
Arsenate	As	0.65930000	-0.07622700	-0.08931900
	S	-1.64029700	0.44365500	-0.76530600
	C	-2.40730000	-0.49336800	0.62115400
	H	-3.47517400	-0.27240000	0.64427400
	H	-2.23694500	-1.55060800	0.41500600
	H	-1.93086100	-0.22474300	1.56420900
	O	0.76422300	-0.61745600	1.60501500
	H	0.89497800	0.20435800	2.11267800
	H	-1.07549800	1.56306100	0.01699900
	O	0.27382100	-1.75996200	-0.59638300
	H	0.73456400	-2.34197900	0.03353400
	O	0.20206200	1.72313800	0.76221400
	H	0.82189600	2.31163800	0.29241300
	O	1.90973100	0.49026700	-0.97255000
MMA^V	As	-0.71495200	0.01662100	-0.08943800
	S	1.81588800	-0.55737700	-0.57948900
	C	2.44527700	0.76058900	0.53934000

	H	3.52787000	0.83541500	0.42693200
	H	1.97680400	1.70192300	0.24511000
	H	2.20719000	0.52713700	1.57937500
	H	1.15944100	-1.42020800	0.43402900
	O	-0.38953000	1.52378600	-1.04782500
	H	-0.38207700	1.26287100	-1.98517900
	O	-0.11489300	-1.49002600	1.12456500
	H	-0.62794200	-2.23242100	0.75297500
	O	-1.79068300	-0.86583700	-0.95236700
	C	-1.05013500	1.03252100	1.52106200
	H	-1.75256200	0.44723000	2.11446700
	H	-0.11544800	1.14906000	2.06989400
	H	-1.46406400	1.99649300	1.22826100
DMA^{V}	As	-0.92069900	-0.00929200	0.04928900
	S	1.99766100	0.66553700	-0.49524500
	C	2.98311700	-0.48800700	0.55140600
	H	4.01098600	-0.52022000	0.17915500
	H	3.00593100	-0.14178800	1.58918400
	H	2.57741300	-1.50424400	0.52747000
	H	1.02935200	-0.61197300	-1.29923500
	O	0.13399800	-1.25676500	-1.21699700
	H	-0.39678400	-1.29113700	-2.03750800
	O	-2.36680000	-0.43487000	-0.59923200
	C	-0.42844000	-0.85422100	1.71107600

	H	-0.14495900	-1.88534100	1.50008000
	H	0.39125900	-0.30283100	2.17069200
	H	-1.32150100	-0.84096200	2.34184800
	C	-0.90578500	1.92821400	0.24669800
	H	-0.74846200	2.37239600	-0.73626200
	H	-1.90597000	2.18666800	0.60814600
	H	-0.10772000	2.21464700	0.92857600

Table S2. The cartesian coordinates for the transition states of Arsenicals reacting with methanol

Arsenical	Atom	Cartesian coordinates		
MMA^{III}	As	-0.36385500	0.12496500	-0.43809100
	C	2.60955400	0.06913000	-0.40313800
	H	2.56436600	0.82651400	-1.20169300
	H	3.56438600	0.19940400	0.12570000
	H	2.62350000	-0.92141100	-0.88879300
	O	-2.05974500	-0.59031200	-0.39927500
	H	-2.54844800	-0.33755800	0.40122500
	O	0.12924600	-1.62977400	0.78600000
	H	-0.46920000	-1.68149800	1.54973200
	C	-0.78397900	1.51506500	0.88938200
	H	0.96853800	-1.03132200	0.99906900
	H	-0.04058900	2.30737600	0.79300600
	H	-1.79006300	1.90613400	0.71507500
	H	-0.70893800	1.09763400	1.89913700
	O	1.54177400	0.22080200	0.50666200
Arsenite	As	0.34622900	-0.22485200	-0.36539000
	C	-2.61343300	-0.13547500	-0.39658600
	H	-2.41771200	0.42392500	-1.32979000
	H	-2.81713300	-1.17906200	-0.67482500
	H	-3.52440900	0.27747100	0.05638700
	O	2.07443700	0.39337700	-0.49117500

	H	2.33166700	0.86902200	0.31776800
	H	-0.95018500	1.15433000	0.80378200
	O	0.70528200	-1.25594000	1.03836400
	H	1.67541800	-1.33001900	1.06549600
	O	0.02054000	1.63878900	0.69655200
	H	-0.04754700	2.33981300	0.02551400
	O	-1.54964200	-0.06654100	0.52788900
Arsenate	As	0.45670400	-0.14197200	-0.07075000
	C	-2.49333600	-0.00980700	-0.03353500
	H	-3.34503500	0.58252000	-0.37557400
	H	-2.60096900	-1.04840700	-0.34225600
	H	-2.39788800	0.05131800	1.05526300
	O	0.49995400	-0.25603700	1.69770200
	H	0.68526000	0.65281600	1.99869200
	H	-0.91520900	1.47815300	-0.29647300
	O	-0.15968600	-1.80669600	-0.19834100
	H	-0.08126700	-2.19652400	0.68905100
	O	0.23887900	1.87656500	0.30796100
	H	0.86819900	2.26798100	-0.32309000
	O	1.69132500	0.04385800	-1.11465200
	O	-1.31101200	0.51181600	-0.67637500
MMA^V	As	0.48230400	-0.09421000	-0.04491300
	C	-2.54184200	-0.14869700	0.02781300
	H	-3.44609000	0.11831900	-0.52639800

	H	-2.42887500	-1.23327500	0.06072300
	H	-2.60968700	0.24943000	1.04756600
	H	-1.04917200	1.39239400	-0.39398100
	O	-0.01207900	-1.83060400	-0.15121900
	H	0.22294300	-2.11418700	-1.05129600
	O	0.10730000	1.91743800	0.10555100
	H	0.65833900	2.27667900	-0.61175600
	O	1.64956100	0.05623500	-1.18081300
	C	0.57942000	-0.10364500	1.88225300
	H	1.40382100	0.55409900	2.15691200
	H	-0.35294000	0.30155500	2.27760300
	H	0.74581900	-1.12973000	2.20628900
	O	-1.40548800	0.38289300	-0.66651100
DMA^{V}	As	-0.52292300	0.06288500	-0.03833400
	C	2.58038400	0.22726600	0.12430600
	H	3.53992000	0.04875800	-0.37465900
	H	2.52092000	1.29136100	0.37683500
	H	2.55674700	-0.36039700	1.05277600
	H	1.17824600	-1.20626000	-0.59965900
	O	0.23098400	-1.89166000	-0.08014100
	H	-0.35400800	-2.26764900	-0.76195800
	O	-1.79560900	-0.34315200	-0.99334200
	C	-0.55826300	-0.10626100	1.89455700
	H	-1.01656700	-1.06178300	2.14462700

	H	0.46129800	-0.06583300	2.28385200
	H	-1.14597600	0.72654500	2.28949600
	C	-0.25723200	1.99076500	-0.27448400
	H	0.00937500	2.16887500	-1.31603200
	H	-1.22280500	2.45729100	-0.06183700
	H	0.50982400	2.37868600	0.39884300
	O	1.51839400	-0.12211600	-0.75571100

Table S3. The cartesian coordinates for the transition states of Arsenicals reacting with methylamine

Arsenical	atom	Cartesian coordinates		
MMA^{III}	As	-0.44619600	0.00734600	-0.45099600
	C	2.52254800	0.47997900	0.00223600
	H	3.40404500	0.29042000	0.62434200
	H	2.63703300	-0.06087000	-0.94065900
	H	2.46155400	1.55230200	-0.20957700
	O	-1.64340200	-0.56482200	0.79249900
	H	-2.11483700	0.21072600	1.13650400
	O	0.28450600	-2.03121000	-0.11408100
	H	-0.37477700	-2.43211700	0.47544000
	C	-0.75100000	1.95996600	0.14201500
	H	1.15497700	-1.10342100	0.63461100
	H	0.00024900	2.59739900	-0.33977300
	H	-1.73197500	2.28014300	-0.23216600
	H	-0.71420100	2.12213800	1.22764000
	N	1.29888600	0.00124200	0.66195600
	H	1.15208000	0.42073900	1.57995300
Arsenite	As	-0.46444000	0.15895900	-0.42440600
	C	2.47049100	0.43069000	0.03912600
	H	2.61465000	0.09352700	-0.99172800
	H	2.35863100	1.51988500	0.03897800
	H	3.35503000	0.15387400	0.62169200

	O	-1.51782800	-0.35746400	0.91940000
	H	-1.41310300	-1.33478100	0.86691700
	H	1.03554300	-1.32498500	0.35786300
	O	-0.57241500	1.90040500	0.07850000
	H	-0.97724000	1.93309000	0.96313100
	O	0.01864900	-2.14679100	-0.09501500
	H	0.17272200	-2.63191500	-0.91914400
	N	1.26177500	-0.20578700	0.57880700
	H	1.09768900	0.03281100	1.55818700
Arsenate	As	0.44220900	-0.16578400	-0.06315800
	C	-2.47798000	-0.06037800	-0.01551100
	H	-3.38001100	0.44088600	-0.37777500
	H	-2.55920000	-1.13941200	-0.16343100
	H	-2.35865900	0.14351200	1.05079500
	O	0.42139900	-0.13372900	1.70598000
	H	0.55030800	0.81682700	1.90065100
	H	-0.84145900	1.54257100	-0.38545200
	O	-0.03046200	-1.88565400	-0.10597300
	H	0.10958000	-2.24097700	0.78744700
	O	0.19932800	1.99820500	0.28982600
	H	0.88250800	2.35956600	-0.29912400
	O	1.68908300	0.06411200	-1.08918900
	N	-1.29131500	0.46689300	-0.71676100
	H	-1.32365200	0.29845700	-1.72364200

MMA^{V}	As	0.44867100	-0.11305500	-0.03055100
	C	-2.52223400	-0.17033500	0.01274800
	H	-3.45163900	0.14819300	-0.46942500
	H	-2.46665100	-1.26244900	0.02777900
	H	-2.52662700	0.20104100	1.04066500
	H	-0.93159300	1.49542000	-0.47752400
	O	0.06930100	-1.89307700	-0.07401500
	H	0.57428000	-2.22856200	-0.83340900
	O	0.16111000	2.02132200	0.06586300
	H	0.77493800	2.32452600	-0.62410700
	O	1.64407500	0.01127200	-1.15033500
	C	0.46239200	0.00025300	1.89844300
	H	1.41959000	0.44837500	2.16907000
	H	-0.33874200	0.66523700	2.21884400
	H	0.36771900	-1.00856200	2.29733400
	N	-1.35730200	0.38778600	-0.69377100
	H	-1.36313100	0.13745800	-1.68391300
DMA^{V}	As	-0.48184300	0.08329800	-0.05056700
	C	2.57393100	0.25996300	0.11390500
	H	2.51064200	-0.19420900	1.10622500
	H	3.53311700	-0.02991300	-0.32889100
	H	2.55578800	1.34949400	0.22380000
	H	1.13068100	-1.33487800	-0.56493200
	O	0.10114500	-2.01902200	0.02323000

	H	-0.51680500	-2.35629300	-0.64682000
	O	-1.65874900	-0.26171200	-1.15050700
	C	-0.58236000	-0.15535400	1.88029300
	H	-1.26596500	-0.98304100	2.06374700
	H	0.40699000	-0.42693000	2.25214600
	H	-0.93630800	0.76716700	2.34673600
	C	-0.29873100	2.04973100	-0.12128800
	H	-0.07816400	2.35611000	-1.14640000
	H	-1.27470600	2.46006300	0.15222400
	H	0.46285300	2.42359000	0.56946300
	N	1.45300300	-0.22194700	-0.70110000
	H	1.50547800	0.09345600	-1.67011700
